# Effects of Gamification in BCI Functional Rehabilitation

**DOI:** 10.3389/fnins.2020.00882

**Published:** 2020-08-21

**Authors:** Martí de Castro-Cros, Marc Sebastian-Romagosa, Javier Rodríguez-Serrano, Eloy Opisso, Manel Ochoa, Rupert Ortner, Christoph Guger, Dani Tost

**Affiliations:** ^1^Universitat Politecnica de Catalunya, Barcelona, Spain; ^2^g.tec medical engineering Spain S.L., Barcelona, Spain; ^3^Guttmann Institute, Badalona, Spain; ^4^g.tec medical engineering GmbH, Schiedlberg, Austria; ^5^Guger Technologies (Austria), Graz, Austria; ^6^Research Center in Biomedical Engineering (CREB), Barcelona, Spain; ^7^Sant Joan de Déu Research Institute, Esplugues de Llobregat, Spain

**Keywords:** brain computer interface, gamification, stroke, rehabilitation, functional rehabilitation, serious game

## Abstract

**Objective:**

To evaluate whether introducing gamification in BCI rehabilitation of the upper limbs of post-stroke patients has a positive impact on their experience without altering their efficacy in creating motor mental images (MI).

**Design:**

A game was designed purposely adapted to the pace and goals of an established BCI-rehabilitation protocol. Rehabilitation was based on a double feedback: functional electrostimulation and animation of a virtual avatar of the patient’s limbs. The game introduced a narrative on top of this visual feedback with an external goal to achieve (protecting bits of cheese from a rat character). A pilot study was performed with 10 patients and a control group of six volunteers. Two rehabilitation sessions were done, each made up of one stage of calibration and two training stages, some stages with the game and others without. The accuracy of the classification computed was taken as a measure to compare the efficacy of MI. Users’ opinions were gathered through a questionnaire. *No potentially identifiable human images or data are presented in this study.*

**Results:**

The gamified rehabilitation presented in the pilot study does not impact on the efficacy of MI, but it improves users experience making it more fun.

**Conclusion:**

These preliminary results are encouraging to continue investigating how game narratives can be introduced in BCI rehabilitation to make it more gratifying and engaging.

## Introduction

Stroke is a leading cause of severe physical disability. According to the World Health Organization, 15 million people suffer from stroke worldwide each year, five million of them die, and five million are permanently disabled (Donkor, 2018). Impairments in the upper limbs affect 60% of stroke survivors. Rehabilitation of these patients is key to improve patients’ capabilities of realizing daily life activities and, consequently, to improve their independence and quality of life (Pindus et al., 2018). Various technologies have been used to support upper limb rehabilitation including assistive robotic systems, camera tracking and motion sensors. Among them, the Mental Imagery Brain Computer Interface (MI-BCI) has emerged as a cost-effective, non-invasive rehabilitation technology, specially indicated for patients with a low range of motor motion, having fatigue, or pain (van Dokkum et al., 2015; Remsik et al., 2016; Cervera et al., 2018).

The strategy of MI-BCI rehabilitation is to exploit the capability of users to create a mental image of a movement. BCI systems use ElectroEncephaloGraphy (EEG) placing electrodes over the patients’ head to capture functional cortical activation changes while patients are trying to create a mental image of a functional motor movement. The EEG signal exhibit event-related synchronization and desynchronization of neural rhythms that can be correlated with the laterality of the mental image (McFarland et al., 2000; Neuper et al., 2006). Thus, machine learning algorithms can be trained to determine in real time if the mental image is correct (Chavarriaga et al., 2017).

Feedback is an essential feature of EEG-BCI rehabilitation. EEG-BCI signal analysis can be used to trigger functional electrostimulation (FES) (Quandt and Hummel, 2014) and to control robotic ortheses in order to assist the realization of motor activity (Ang et al., 2015). In this way, the disrupted sensorimotor loop is closed. It has been proven that this loop closure is a key factor to induce neural plasticity changes, therefore to improve functional behavior. Visual feedback is necessary to learn how to create mental images. In addition, during the routine use of BCI, it provides users with self-awareness and assessment of how they are performing. The suitability of different forms of feedback has been discussed (Lotte et al., 2013; Jeunet et al., 2016). On one hand, symbolic widgets such as progress bars and arrows are simple and fast to implement, but they have been found to be difficult to understand and may even distract users (Kosmyna and Lécuyer, 2017; Škola et al., 2019). On the other hand, embodied avatar representations of the patient’s limb promote Action Observation mechanisms and activate the Mirror Neuron Network (MNN) inducing thus cortical plasticity (Pichiorri et al., 2015; Zhang et al., 2018). Moreover, the sense of embodiment that a realistic avatar provides impacts positively on BCI control (Petit et al., 2015; Alimardani et al., 2016).

BCI sessions are based on repetition of exercises, they are cognitively demanding and can lead to a reduced patient engagement in rehabilitation. Gamification is defined as the introduction of game-design elements and principles such as narratives, scores and awards in non-game contexts to increase a person satisfaction and interest in performing activities by bringing intrinsically motivational playful experiences (Richter et al., 2015). Gamification has become a popular research topic with applications in a variety of domains from corporate business transformation to education and health (Zichermann and Linder, 2013). However, some studies in domains such as education, have shown that it is not always effective. Moreover, it can even yield to a reduction of the efficacy of the activity it aims at making more motivating (Hamari et al., 2014). The effects of gamification are greatly dependent on the context and on the users. In particular, rewards, badges and leaderboards should be used with precaution as they may backfire (Hanus and Fox, 2015).

Gamification has been largely used in conventional upper-arm rehabilitation in order to alleviate the repetitiveness of sessions, increase motivation, and engagement (Burke et al., 2009; Bermúdez-Badia et al., 2016). Commercial computer games have been adapted and new games have been designed on purpose to enhance the rehabilitation experience (Bermúdez-Badia and Cameirão, 2012). These games use the movement of the patients as the input system of the game. The movement is measured through various tracking systems (Llorens et al., 2015), and it substitutes conventional devices such as mouse and joysticks.

The introduction of gamification in BCI rehabilitation is quite challenging because using brain signals as the only user input reduces the scope of possible game narratives. Moreover, in order to keep the benefits of embodiment (Borrego et al., 2019), games should somewhat integrate the patient’s upper limb avatar. This is why existing studies typically involve driving or navigation tasks: for instance, destroying asteroids using left/right hand (Vourvopoulos et al., 2016) or rowing boats while trying to collect flags (Vourvopoulos et al., 2019). Existing gamified BCI solutions have been basically tested with volunteer participants that have not been affected by a stroke, thus there is a lack of data on actual patients. Little is known about the impact of introducing external stimuli such as game elements aside from the avatar’s limb on the efficacy of the training activity.

In this paper, we present a preliminary experimental study on gamified BCI post-stroke functional rehabilitation of the upper limbs. The goal of the study is to analyze how gamification impacts on the efficacy of the treatment and on patients’ experience.

## Materials and Methods

### Setup

The BCI system used on this study is recoveriX^®^ (g.tec medical engineering GmbH, Austria). The system analyzes the EEG brain signals and provides multimodal feedback through a virtual reality avatar of the upper limbs and a FES proprioceptive feedback stimulation (Irimia et al., 2016, 2017; Cho et al., 2016). The EEG caps were equipped with 16 active electrodes (g.LADYbird or g.Scarabeo, g.tec medical engineering GmbH) located according to international 10/10 system (extended 10/20 system): FC5, FC1, FCz, FC2, FC6, C5 C3, C1, Cz, C2, C4, C6, Cp5, Cp1, Cp2, Cp6. A reference electrode was placed on the right earlobe and a ground electrode at position of Fpz.

### Game Design

**FIGURE 1:**
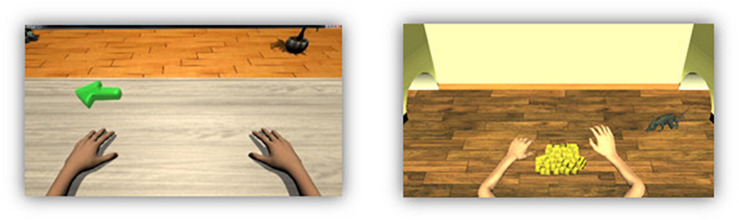
Standard avatar and new game appearance. In the left side, is the avatar used in recoveriX system, the green arrow indicates in which hand the movement should be performed. In the right side is the new animated game, both arms are in the same position than the standard avatar. In front of the virtual subject there are 80 pieces of cheese that the user should try to keep. The rat indicates which hand should move.

The game was developed on top of this system with two main requirements. First, it could not alter the pace of the rehabilitation. Second, in order to avoid altering the sense of identification of the user with the virtual forehand, the game could not modify the gesture of the avatar. With these limitations, the narrative was restricted to a game in which the unique action of the avatar was raising and lowering the wrist. Moreover, to make the virtual situation as similar as possible to the real one, we avoided driving-like actions that imply a virtual navigation of the avatar. We also wanted to have feedback of the current exercise and of the total training stage so far. Hence, the goal of the game is to compete with a mouse in order to preserve food. Figure 1 shows the “standard” avatar and the new game appearance. At the beginning of the session 80 pieces of cheese (one for each exercise) are set between the two virtual arms. At each exercise, a mouse appears from the right or left corner of the room (the side of the wrist that must move) and stands nearby the pile of cheese pieces during the cue sub-stage. In the feedback sub-stage, the game receives a cue of Boolean events that indicate if the mental image is being correct or not. The avatar’s hand moves accordingly, and the FES is activated. When a cue is incorrect, both the visual feedback and the electrical stimulation are disabled. In the relax sub-stage, if five consecutive events are considered correct, when the virtual arm lowers, the mouse runs away empty-handed. Otherwise, it takes a piece of cheese. The size of the pile is thus an indicator of the overall progress of the training stage. In addition, a scoring panel was added to reinforce the awareness of the user. This panel could be deactivated, shown intermittently or constantly displayed. The game was implemented with Unity and connected to the recoveriX^®^ replacing the non-gamified version. It is available upon request by mail to the corresponding author.

### Participants

Ten stroke patients with hemiparesis in the upper limb and six healthy subjects were recruited for this study. The stroke subjects were patients from Institut Guttmann. All participants were volunteers. The inclusion criteria for stroke patients were: (i) residual hemiparesis, (ii) the stroke occurred at least 4 days before the first assessment, (iii) functional restriction in the upper extremities. Additionally, for all participants, the following criteria were applied: (iv) to be able to understand written and spoken instructions, (v) stable neurological status, (vi) willing to participate in the study and to understand and sign the informed consent, (vii) to be able to attend meetings. Ethics approval was obtained from the Ethic committee of Institut Guttmann, Barcelona, Spain. Finally, all participants were informed about the goals of the project, and they provided their written informed consent before participating in the study.

### Experimental Design

**FIGURE 2:**
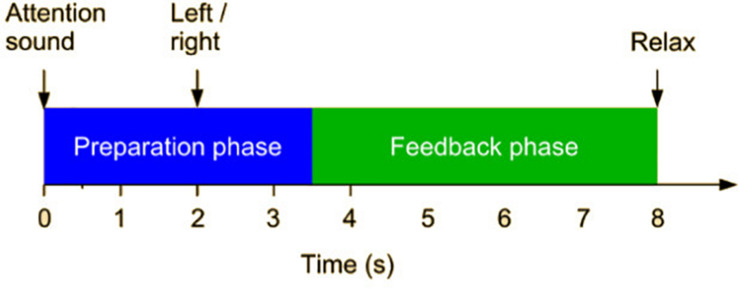
RecoveriX trial description.

All participants took part in the same procedure: control users in the research lab and patients in the rehabilitation institution. They performed two training sessions separated in time by a minimum of 1 day and a maximum of 2 weeks. Each session was composed of three runs or stages: Calibration (C-S1, C-S2), Training 1 (T1-S1, T1-S2), and Training 2 (T2-S1 and T2-S2). Each run was composed of 80 trials (80 movements) and lasted 12 min. There was a resting time of about 5 min between stages. Figure 2 describes the timing of each trial. Each movement started with a cue, and 2 s later the system presented an arrow pointing to the movement direction. The participant was instructed to start the MI just after the cue for the next 6 s. During this period the user had to imagine the wrist dorsiflexion, and the feedback devices were activated. After the feedback period the system provided a sound to mark the end of the exercise and gave 2 s of rest before the next trial.

### Motor Imagery Accuracy Calculation

The EEG data was bandpass filtered (0.5–30 Hz) to increase the signal to noise ratio (SNR) and to remove unnecessary components. We also applied a 50 Hz notch filter to reduce line noise. We then created 8 s epochs of EEG data for every trial and divided them into two classes: left and right.

Each epoch was bandpass filtered (8–30 Hz) and an artifact rejection was applied (the same as in the lateralization coefficient). Using the current frames, a CSP filter was created. Next, it was used to get 4 spatially filtered channels from the 16 EEG channels. For every frame we defined 14 timepoints, separated 0.5 s one from each other, from 1.5 to 8 s of the frames. For each timepoint we calculated a set of 4 features.

For each timepoint, we calculated the variance of each spatially filtered signal using a window of 1.5 s. The resulting four features for each timepoint were normalized, and we then derived their logarithmic values. Using all the features from all the timepoints and the entire frame collection, we calculated a linear discriminant analysis (LDA) classifier.

Using the CSP filter and the LDA classifier, the classifier accuracy is assessed with a 10-fold cross validation process. During this process, a classifier is created for every fold using 90% of the frames (training set). The classifier is then assessed with the other frames (testing set). This is done 10 times, and ultimately yields a mean accuracy for each class (left and right hand) and every timepoint. Finally, for each class, the MI accuracy is calculated as the maximum (Max. Accuracy) or as the mean (Mean Accuracy), among all timepoints. The LDA classifier was not modified from the original version (Irimia et al., 2016) to support the gamification pilot. Its code is not publicly available.

The calibration run is used to train the LDA classifier, thus, during this run the online feedback provided to users is always positive. After the calibration run, all participants were moved to the “Training” mode, where the feedback is triggered by the MI in real time. During Training 1 feedback is based on the classifier built after Calibration, and during Training 2 it is based on an enhanced version of the classifier using data from the previous two stages. Each session started from scratch; thus Session 2 did not use the classifier of Session 1.

During the two sessions subjects sat at a table with the computer screen in front. They wore headphones to listen to the instructions and sounds.

In the first session, calibration (C-S1) and Training 1 (T1-S1) were without the game, only with the regular avatar, while Training 2 (T2-S1) used the game without any feedback of time and scoring *(no feedback)*. In the second session, all stages used the game: C-S2 (*no feedback)*, T1-S2 showing score and time every ten exercises *(intermittent feedback)* and T2-S2 showing time and score constantly *(constant feedback)*.

The feedback received by the users is shown in Figure 1. As mentioned, there are two kinds of feedback: time and score. The time is shown through a cheese-shape clock while the score is shown literally differentiating the user score, under the name of Jasper, and the rat score.

### Assessment Test

For this study two variables were analyzed: BCI performance and users’ experience. BCI performance was studied using the MI accuracy of each run computed as exposed above. Users’ experience was assessed using a questionnaire.

### Questionnaire

The opinions of users about the game were gathered through a customized version of the System Usability Scale (SUS) composed by 8-items to be answered in a Likaert scale of 1–5, being 1 the worst case and 5 the best (see Table 1).

**TABLE 1:** Users’ experience questionnaire.

#	Question	Score
Q1	Evaluate the level of fun in the game.	[1] no fun; [2] little fun; [3] indifferent; [4] fun; [5] very fun
Q2	Evaluates the visual aspect of the game.	[1] very bad; [2] bad; [3] indifferent; [4] good; [5] very good
Q3	Evaluate the easeiness of use of the game.	[1] very hard; [2] hard; [3] normal; [4] easy; [5] very easy
Q4	Evaluate the clarity of rules of the game.	[1] very confusing; [2] confusing; [3] indifferent; [4] clear; [5] very clear
Q5	With regard to the narrative plot (the fight against the mouse to protect the cheese), you thought so.	[1] very inadequate; [2] inadequate; [3] indifferent; [4] adequate; [5] very adequate
Q6	With regard to the level of concentration required to perform the exercise, in your opinion, adding the game to the rehabilitation session has contributed to:	[1] has distracted me a lot; [2] has distracted me; [3] has not influenced me; [4] has helped me to concentrate; [5] has helped me to concentrate a lot
Q7	With regard to possible boredom while exercising, in your opinion, adding the game to the rehabilitation session has contributed to:	[1] It’s increased a lot more boredom; [2] It’s bored me more; [3] It has not influenced me; [4] It alleviated boredom more; [5] It alleviated boredom a lot more
Q8	In general, the idea of introducing a game (not necessarily this one) into rehabilitation therapy, seems:	[1] very bad; [2] bad; [3] indifferent; [4] good; [5] very good

In addition, all participants were asked about how often they played videogames in a 5-values scale (never, sometimes, often, usually, always), and if they had previous experience with BCI technology. The answers and all collected data are available at the git repository: https://github.com/nosepas1/BCI_gamification_data.

### Statistical Analysis

The software used for the statistical analysis was MATLAB R2017a and a python script using scipy stats, numpy and pandas. The first step of the statistical analysis is the comparison of the baselines of each group of participants; age, gender, and precision. First, the Shapiro-Wilk Test (SWT) test was performed to analyze the normality of the variables. For the comparison between groups (“Healthy” and “Stroke”), *t*-test for independent samples (in case of assumption of normality) and Mann–Whitney *U* test (in case of non-normality) were used.

For the analysis of the impact of the serious game combined with BCI on the user’s concentration, since no independence could be assumed, the MI accuracies of every subject in all games mode were compared. The selected test for the analysis was “repeated measures ANOVA” (Girden, 1992; Norman and Streiner, 2008; Singh et al., 2013; Verma, 2015), which allows the results’ comparison of the same group of participants at different time points. For that, two assumptions are needed: normality distribution (Shapiro-Wilk test > 0.05) and assumption of sphericity (Mauchly’s sphericity test > 0.05).

Finally, a quantitative analysis of the answers in the questionnaire of each participant was carried out.

## Results

### Participants Baseline

Six healthy subjects and ten stroke patients were enrolled in the study, seven of them were females and nine males. The average age of the healthy group was 35.3 years old (*SD* = 16.0), with the maximum and minimum age in this group was 58 and 23 years old, respectively. The mean age of the stroke group was 55.8 years old and the maximum and minimum age was 79 and 26 years old. In the Stroke group, four patients had been affected on their right side, and 6 on their left side. The mean time since stroke was 33 months (*SD* = 22.8), seven in subacute phase, Three in chronic phase, and 0 in acute phase. Neither patients nor control users had previous experience in BCIs, except two patients that had used the recoveriX^®^ system years ago. Control users had neither previous known neurological disorder, nor previous experience in BCIs.

The accuracy obtained after the first training run in the first session (T1-S1) is taken as a baseline reference for each subject. As mentioned above, in run T1-S1, participants used the standard visual feedback with a personalized classifier generated in the calibration run of Session 1(T1-C1). Thus, the accuracy obtained in T2-S1, T1-S2, and T2-S2 is compared with that of T1-S1. The equality of the baselines cannot be assumed, because there is a statistical difference in the age between groups. The age variable of the healthy group is not normally distributed (SWT: *P* = 0.022) and Mann-Whitney *U* test shows a significant difference between both age groups, *P* = 0.031. In order to see how much the age differences can influence the BCI performance, the correlation between the age and the maximum classification accuracy (maximum accuracy of the second run in the first session T1-S2) has been studied. The age variable with all participants and MI accuracy data follow a normal distribution (SWT age, *P* = 0.075, SWT accuracy, *P* = 0.096). The Pearson correlation test shows that there is no significant correlation between age and accuracy (rho = −0.195, *P* = 0.505). Thus, the comparison of the MI accuracy between groups is allowed. However, because of the small size of sample no general conclusion can be extracted about the relationship age and accuracy.

The comparison of the accuracy obtained in the first training run T1-S1 (after system calibration), shows that there is no statistical difference in the BCI performance between healthy and stroke group using unpaired *t*-test, *t*-value = |1.475| and *P* = 0.166 (SWT > 0.05).

### Impact of the Game in the BCI Performance

In order to detect differences in the accuracy using different visual feedback modalities, the MI accuracy of each run has been analyzed using repeated measures ANOVA. All the datasets can be considered normally distributed. Shapiro-Wilk test did not show significant results at alpha level. Mauchly’s Test of Sphericity indicated that the assumption of sphericity has not been violated, χ2(2) = 9.595, *P* = 0.088.

**FIGURE 3:**
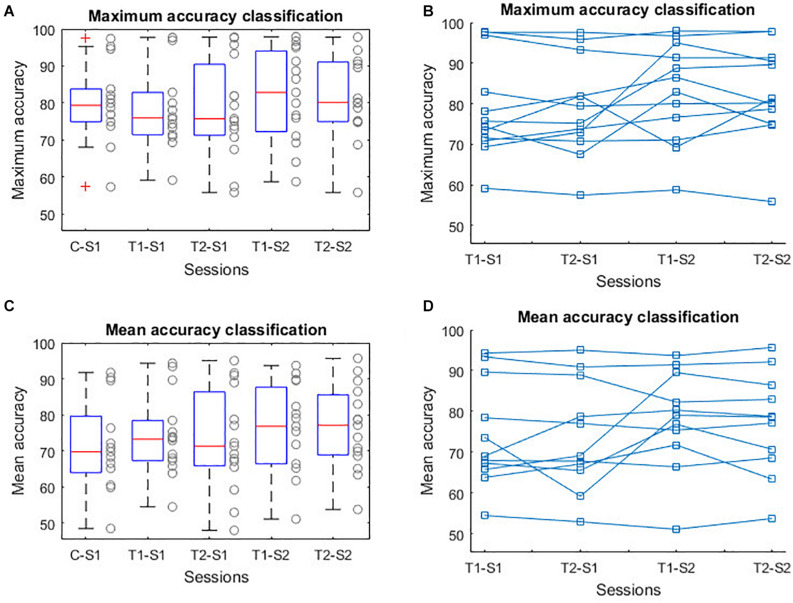
BCI performance using different visual feedback.

Table 2 shows the results of the accuracy comparison using repeated measures ANOVA. The multiple comparison did not show statistical differences in the accuracy based on the gamification with different visual feedback modalities (see Figure 3 and Table 3). The same comparison has been done using only the data from the healthy or stroke group, and no significant differences have been detected.

**TABLE 2:** Multiple comparison of MI accuracy using repeated measures ANOVA.

	SumSq	*df*	MeanSq	*F*	*p*-Value	*p*-ValueGG	*p*-ValueHF	*p*-ValueLB
**Maximum accuracy**								
Intercept	72.272	2	36.136	1.4213	0.265	0.266	0.266	0.261
run2_ses1	49.236	2	24.618	0.96826	0.397	0.374	0.382	0.348
Error	508.50	20	25.425					
**Mean accuracy**								
Intercept	72.508	2	36.254	1.3514	0.284	0.280	0.281	0.275
run2_ses1	48.422	2	24.211	0.90249	0.423	0.385	0.392	0.367
Error	482.88	18	26.827					

**TABLE 3:** Summary of MI accuracy of each group.

	C-S1	T1_S1	T2_S1	T1_S2	T2_S2
**Maximum accuracy**					
All (mean)	80.09 (10.76)	78.86 (11.47)	78.49 (13.32)	82.03 (12.48)	82.08 (11.61)
Healthy (mean)	84.78 (12.9)	85.70 (14.25)	83.68 (16.9)	86.42 (15.39)	88.42 (11.41)
Stroke (mean)	78.21 (9.91)	76.12 (9.65)	75.02 (9.9)	79.11 (10.02)	77.86 (10.22)
**Mean accuracy**					
All (mean)	71.53 (12.82)	74.29 (11.47)	73.07 (14.15)	76.09 (12.33)	76.45 (11.63)
Healthy (mean)	80.40 (17.77)	81.86 (14.26)	77.84 (18.17)	81.93 (14.37)	83.77 (11.83)
Stroke (mean)	68.87 (10.71)	71.27 (9.31)	69.88 (10.75)	72.20 (9.73)	71.57 (9.07)

While no significant differences are shown in several ANOVA tests, from inspection of Figure 3, a trend toward an improvement of mean accuracy along the sessions seems plausible. However, no conclusive results can be drawn because of the small number of subjects.

### Users’ Satisfaction With the Serious Game

The users’ satisfaction was assessed after the last session using a questionnaire with eight questions rated from 1 to 5. For the quantification of the results the average of the individual score and the average of each question in the questionnaire has been computed.

**FIGURE 4:**
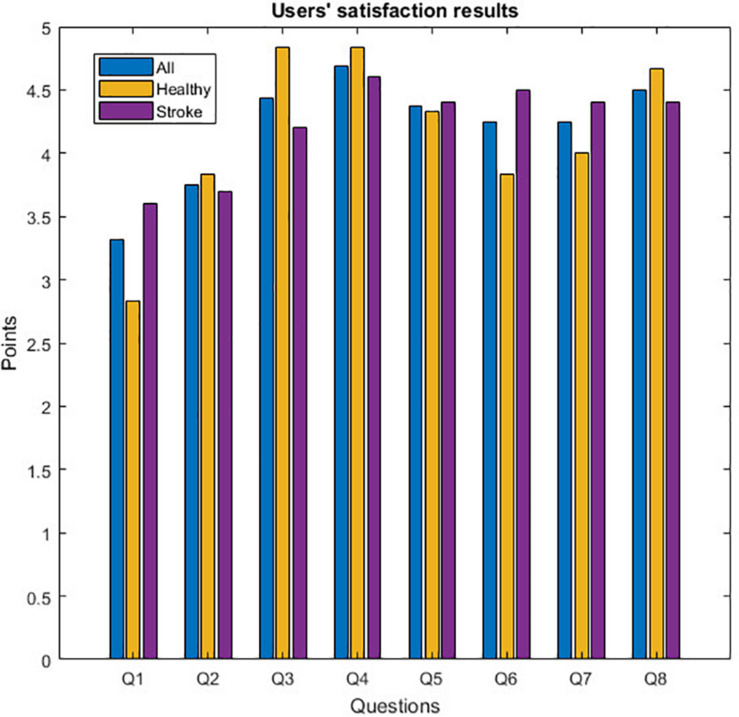
Questionnaire results.

Table 4 shows the results in the questionnaire based on groups and gaming experience. The first column shows the group name, the second column the group size, the third column is the averaged total questionnaire score based on the average score in each question, and the next eight columns show the average result for each group of each question. Figure 4 shows the questionnaire results of each group.

**TABLE 4:** Summary of questionnaire results based on group and gaming experience.

	n	Mean (*SD*)	P1	P2	P3	P4	P5	P6	P7	P8
**All**	**16**	**4.20 (0.45)**	**3,31**	**3,75**	**4,44**	**4,69**	**4,38**	**4,25**	**4,25**	**4,50**
**Healthy**	**6**	**4.15 (0.68)**	**2,83**	**3,83**	**4,83**	**4,83**	**4,33**	**3,83**	**4,00**	**4,67**
Often	3	4.54 (0.56)	3,33	4,33	5,00	5,00	4,67	4,33	4,67	5,00
Sometimes	2	3.81 (0.80)	2,50	3,50	4,50	4,50	4,50	3,50	3,00	4,50
Never	1	3.63 (1.06)	2,00	3,00	5,00	5,00	3,00	3,00	4,00	4,00
**Stroke**	**10**	**4.23 (0.37)**	**3,60**	**3,70**	**4,20**	**4,60**	**4,40**	**4,50**	**4,40**	**4,40**
Often	1	3.75 (1.04)	3,00	2,00	4,00	4,00	3,00	5,00	4,00	5,00
Sometimes	3	4.13 (0.56)	3,00	3,67	4,00	4,33	4,67	4,67	4,33	4,33
Almost never	1	3.13 (0.83)	3,00	2,00	2,00	4,00	4,00	3,00	4,00	3,00
Never	5	4.6 (0.24)	4,20	4,40	4,80	5,00	4,60	4,60	4,60	4,60

*The bold values differentiate between the two groups of users that participate in the experiment. People that have suffer the stroke and healthy people.*

All participants gave high scores in all questions: users’ satisfaction is 4.20 points (*SD* = 0.45) up to five, the stroke group gave higher score in the questionnaire with 4.23 points (*SD* = 0.35), whereas the healthy group was 4.15 points (*SD* = 0.63). In general, the best aspect of the game is the clarity of the rules (Q4). The healthy group also highlighted the easiness of use (Q3). The worst aspect is the fun level of the game (Q1). In the informal debriefing after the sessions, users declared being pleased with the game, but suggested some enhancements such as introducing variations in the animation of the rat, which is always the same, and adding new auditory stimuli. The attention and somnolence in stroke patients are always a problem, which is not always discussed and should be considered in the design of experiments. In this case, patients agreed that the activity had the proper duration to avoid these problems. Stress was not quantitatively measured. However, in the debriefing session, patients did not mention any change in the level of fatigue and stress using the gamified version of training.

Finally, no significant correlation was found between the questionnaire score and accuracy.

## Discussion

The objective of this experiment was to explore how the proposed serious game can affect users’ concentration and performance of a BCI system for stroke functional rehabilitation.

Although the Healthy and Stroke groups presented significant differences in age, this unevenness does not seem to harm the analysis, because there is no lineal correlation between age and accuracy (Pearson’s test; rho = −0.195, *P* = 0.505). However, the number of subjects is too small to generalize this conclusion. In future experiments, with more subjects, ages will be stratified. Furthermore, there was no differences in the MI accuracy between the Healthy group and the Stroke group (*t*-test, *t*-value = |1.475| and *P* = 0.166).

The BCI performance has been studied through a multiple comparison analysis using the MI accuracy calculated after each run using different avatar versions. The comparison using repeated measures ANOVA test, showed no significant results, in the mean accuracy as well as in the maximum accuracy (Tables 2, 3 and Figure 3). The results of this first analysis demonstrate that there is no negative effect in the BCI performance when it is combined with a new gamified avatar. However, as shown in Figure 3A, the point cloud of T1-S2 and T2-S2 are slightly higher than T1-S1 (MI accuracy baseline measure). This difference is more evident in the mean accuracy plot (Figure 3C). The most probable explanation for that is that the pop-up scoring window can encourage the user to be more focused in the MI task.

The results obtained from the questionnaire show a high satisfaction level from the users (see Figure 4). In one hand, the easiness of use and the clarity of the rules are the features best scored by both groups. It is important to point out that previous experience on gaming is not related with better user experience or a better BCI performance. All users also reported that this new avatar helped them to improve their concentration (Q6) and reduce their boredom (Q7). This is consistent with the results obtained in Figure 3C. On the other hand, all participants gave the lowest score to the entertainment level (Q1) and visual attractiveness (Q2). As observed in previous experiments (Lledó et al., 2016), visual attractiveness is a desired objective but sometimes patients prefer simpler versions of a task. Future versions of the game could provide different versions of the game appearance. The difficult part is to improve the entertainment level of the game without increasing the cognitive task and, consequently, decreasing the BCI performance. Hence, other narrative threads could be tested and stratified into levels to assess how a story impacts on users’ performance and motivation. Moreover, the game difficulty level could be adapted to the user’s performance: the better the results, the higher the correct response threshold.

The main limitation of the study is small number of subjects and the age difference between groups. In addition, more sessions are needed to evaluate if the results observed in this pilot study are generalizable. Furthermore, new variables can be considered such us stress and fatigue, frequent in this type of rehabilitation. Finally, some emotional variables can be included to compare with the user performance.

Nevertheless, the idea of introducing games combined with BCI therapy seems to be an promising step to take to improve user experience, increase adherence to treatment and improve the functional outcome of patients.

## Conclusion

A game-based rehabilitation instrument has been developed as an improvement of the existing recoveriX system for post-stroke upper limb rehabilitation. A pilot study has been carried out to test the impact of the game in the rehabilitation process. Sixteen subjects were recruited (6 healthy and 10 stroke patients) to perform 2 sessions of BCI therapy using different visual feedback modalities. The first run (80 trials) of each session was used to calibrate the system creating a personal LDA classifier. In the second run of the first session (T1-S1) all participants performed 80 trials using the “standard” VR avatar. In the third run of the first session (T2-S1) the participants used a new animated version based on the standard avatar. In the second run of the second session (T2-S2) users trained with the new avatar combined with a pop-up window that was appearing for a short period every 10 min showing the score. In the third run of the second session (T2-S2) the appearance was like the T2-S2, but the score window was appearing all the time. The objective of these last two runs was to add more cognitive responses to improve the concentration without harming the MI accuracy.

The results show there is no significant difference in the MI accuracy baseline between the healthy group and the stroke group. Moreover, there were no significant differences either between training with or without game. Results also show that there are no significant differences in the accuracies using the different forms of scoring feedback. Thus, the added stimuli of scoring and time does not affect performance. Concerning users’ opinions, they were all positive about the game level of entertainment, clarity of rules, narrative and visual attractiveness. Participants declared not having been affected by the game to create a mental image but having felt less bored. Finally, there was a consensus about the interest of gamifying stroke rehabilitation sessions. The main limitation of this study is the small size of the sample and small number of rehabilitation sessions. However, the results are encouraging to continue investigating how to bring gamification elements to post-stroke rehabilitation.

## Data Availability Statement

The datasets generated for this study are available on request to the corresponding author.

## Ethics Statement

The studies involving human participants were reviewed and approved by the Institut Guttmann ethics committee. The patients/participants provided their written informed consent to participate in this study. Written, informed consent was obtained from the individuals for the publication of any potentially identifiable images or data included in this article.

## Author Contributions

MC-C contributed to the implementation and execution of the methodology and writing the manuscript. MS-R collaborated in the design of the experiment, the analysis of the results, and writing the manuscript. JR-S participated in the design of the experiment and in the set up of the brain computer interface, and the integration of the game. EO and MO collaborated in the organization of the pilot study with patients. EO reviewed the manuscript. RO and CG contributed to the design of the BCI technology. DT supervised the research and contributed to the design of the game, the analysis of the results, and writing the manuscript. All authors contributed to the article and approved the submitted version.

## Conflict of Interest

MC-C, MS-R, JR-S, and RO were employed by the company g.tec medical engineering Spain S.L. CG is CEO of the company g.tec medical engineering Spain S.L. and g.tec medical engineering GmbH. The remaining authors declare that the research was conducted in the absence of any commercial or financial relationships that could be construed as a potential conflict of interest.
