# Supplementary material for: Effects of Gamification in BCI Functional Rehabilitation
Source: Front Neurosci. 2020 Aug 21;14:882. doi: 10.3389/fnins.2020.00882 (PMC7472985; doi:10.3389/fnins.2020.00882)
Supplement: Supplementary file 1 [file Table_1.DOCX]

**Table 1:** Accuracies and significance of control users (CU1 to CU6) and patients (TU1 to TU10) during the three stages of training in the two sessions.

|  | First session | | | | | | | | | Second session | | | | | | | | | |
| --- | --- | --- | --- | --- | --- | --- | --- | --- | --- | --- | --- | --- | --- | --- | --- | --- | --- | --- | --- |
|  | C-S1 | | | T1-S1 | | | T2-S1 | | | C-S2 | | | T1-S2 | | | T2-S2 | | | |
|  | Max | Mean | S | Max | Mean | S | Max | Mean | S | Max | Mean | S | Max | Mean | S | | Max | Mean | S |
|  | CONTROL USER | | | | | | | | | | | | | | | | | | |
| CU1 | NaN | NaN | NaN | NaN | NaN | NaN | 97,8 | 91,6 | 65,1 | NaN | NaN | NaN | 95,5 | 89,8 | 59,5 | | 94,3 | 89,4 | 58 |
| CU2 | 68,1 | 59,9 | 62 | 69,4 | 65,8 | 59,1 | 73,0 | 69,2 | 57,5 | 90,3 | 85,9 | 62 | 95,0 | 89,5 | 63,2 | | 90,5 | 86,5 | 61,9 |
| CU3 | NaN | NaN | NaN | NaN | NaN | NaN | 55,9 | 48,0 | 58,2 | 74,1 | 68,0 | 63,4 | 64,1 | 61,8 | 60,6 | | 68,8 | 65,1 | 58,6 |
| CU4 | 95,3 | 91,7 | 62,8 | 97,6 | 94,3 | 59 | 97,6 | 95,0 | 57,9 | 94,7 | 92,9 | 61,7 | 96,7 | 93,7 | 58,2 | | 97,8 | 95,7 | 56,6 |
| CU5 | 81,1 | NaN | 61,9 | 78,1 | 73,9 | 59,5 | 82,0 | 72,3 | 58,2 | 65,2 | 61,5 | 62,6 | 69,3 | 65,3 | 60,9 | | 81,3 | 73,8 | 58,7 |
| CU6 | 94,6 | 89,5 | 63,7 | 97,7 | 93,4 | 58,8 | 95,8 | 90,9 | 57,3 | 92,3 | 79,5 | 61,5 | 97,9 | 91,5 | 58,4 | | 97,8 | 92,2 | 56,7 |
| AVG CU | 84,8 | 90,6 | 62,6 | 85,7 | 81,9 | 59,1 | 89,2 | 83,8 | 59,0 | 83,3 | 81,6 | 62,2 | 86,4 | 81,9 | 60,1 | | 88,4 | 83,8 | 58,4 |
| STD CU | 12,9 | 1,6 | 0,8 | 14,2 | 12,5 | 0,3 | 11,2 | 12,1 | 3,0 | 13,0 | 10,6 | 0,8 | 15,4 | 14,4 | 1,9 | | 11,4 | 11,82 | 1,9 |
|  | PATIENTS | | | | | | | | | | | | | | | | | | |
| TU1 | 73,8 | 65,2 | 61,4 | 71,5 | 68,0 | 58 | 70,8 | 67,9 | 56,5 | 64,1 | 59,2 | 61,5 | 71,1 | 66,4 | 58,2 | | 74,8 | 68,6 | 56,6 |
| TU2 | 83,8 | 68,3 | 61,9 | 75,7 | 73,6 | 58,2 | 75,2 | 59,3 | 56,6 | 82,9 | 74,2 | 61,7 | 88,7 | 79,1 | 58,5 | | 89,6 | 78,6 | 56,9 |
| TU3 | 82,1 | 76,4 | 61,5 | 73,4 | 69,1 | 58 | 81,9 | 78,8 | 56,5 | 83,8 | 76,3 | 61,4 | 86,5 | 80,3 | 58,1 | | 80,0 | 78,8 | 56,6 |
| TU4 | 76,9 | 69,8 | 61,5 | 74,4 | 67,3 | 58 | 67,5 | 65,5 | 56,5 | 82,9 | 75,4 | 61,7 | 82,9 | 76,9 | 58 | | 75,0 | 70,8 | 56,6 |
| TU5 | 76,9 | 72,2 | 61,5 | 82,9 | 78,5 | 58,2 | 79,4 | 77,1 | 56,5 | 79,5 | 72,9 | 61,5 | 80,0 | 75,4 | 58,2 | | 80,2 | 77,2 | 56,6 |
| TU6 | 97,4 | 90,3 | 61,5 | 96,9 | 89,6 | 58 | 93,3 | 88,9 | 56,5 | 91,3 | 82,0 | 61,4 | 91,3 | 82,3 | 58 | | 91,3 | 83,0 | 56,5 |
| TU7 | 57,4 | 48,5 | 62,4 | 59,2 | 54,5 | 58,5 | 57,5 | 53,0 | 57,1 | 64,3 | 59,2 | 62,2 | 58,8 | 51,2 | 58,3 | | 55,9 | 53,8 | 56,7 |
| TU8 | 78,8 | 66,6 | 61,4 | 70,9 | 63,8 | 58 | 73,8 | 67,1 | 56,5 | 83,3 | 70,5 | 61,5 | 76,7 | 71,8 | 58,2 | | 78,7 | 63,4 | 56,6 |
| TU9 | 75,0 | 60,5 | 61,4 | 76,3 | 72,9 | 58,1 | NaN | NaN | 0,0 | 81,4 | 68,6 | 62,2 | 76,0 | 66,4 | 58,3 | | 75,2 | 69,9 | 56,7 |
| TU10 | 80,0 | 70,9 | 61,4 | 80,0 | 75,3 | 58 | 75,8 | 71,4 | 56,5 | 69,7 | 62,0 | 61,7 | NaN | NaN | NaN | | NaN | NaN | NaN |
| AVG TU | 80,5 | 72,5 | 61,6 | 76,1 | 73,1 | 58,1 | 75,0 | 72,0 | 56,6 | 78,3 | 72,7 | 61,7 | 79,1 | 74,8 | 58,2 | | 80,6 | 73,8 | 56,6 |
| STD TU | 7,1 | 7,99 | 0,3 | 9,7 | 7,7 | 0,2 | 9,9 | 9,3 | 0,2 | 9,12 | 7,6 | 0,3 | 10,0 | 6,1 | 0,2 | | 6,5 | 6,6 | 0,1 |

**Table 2:** Answers to the questionnaires of control and patient users

| User | Genre | age | Game profile | Q1 | Q2 | Q3 | Q4 | Q5 | Q6 | Q7 | Q8 |
| --- | --- | --- | --- | --- | --- | --- | --- | --- | --- | --- | --- |
| CU1 | F | 22 | Sometimes | 3 | 4 | 4 | 4 | 5 | 3 | 2 | 4 |
| CU2 | M | 23 | Often | 4 | 4 | 5 | 5 | 4 | 3 | 4 | 5 |
| CU3 | M | 26 | Often | 2 | 4 | 5 | 5 | 5 | 5 | 5 | 5 |
| CU4 | M | 25 | Sometimes | 2 | 3 | 5 | 5 | 4 | 4 | 4 | 5 |
| CU5 | F | 53 | Never | 2 | 3 | 5 | 5 | 3 | 3 | 4 | 4 |
| CU6 | F | 57 | Often | 4 | 5 | 5 | 5 | 5 | 5 | 5 | 5 |
| TU1 | M | 41 | Never | 4 | 4 | 5 | 5 | 5 | 5 | 5 | 5 |
| TU2 | M | 78 | Never | 3 | 3 | 4 | 5 | 3 | 4 | 4 | 3 |
| TU3 | M | 57 | Never | 5 | 5 | 5 | 5 | 5 | 5 | 5 | 5 |
| TU4 | M | 53 | Never | 5 | 5 | 5 | 5 | 5 | 5 | 5 | 5 |
| TU5 | F | 25 | Never | 4 | 5 | 5 | 5 | 5 | 4 | 4 | 5 |
| TU6 | F | 61 | Often | 3 | 2 | 4 | 4 | 3 | 5 | 4 | 5 |
| TU7 | M | 67 | Almost never | 3 | 2 | 2 | 4 | 4 | 3 | 4 | 3 |
| TU8 | F | 57 | Sometimes | 4 | 5 | 4 | 5 | 4 | 5 | 5 | 5 |
| TU9 | F | 60 | Sometimes | 3 | 2 | 3 | 3 | 5 | 4 | 3 | 3 |
| TU10 | M | 51 | Sometimes | 2 | 4 | 5 | 5 | 5 | 5 | 5 | 5 |
